# Identification of Simple Sequence Repeat Biomarkers through Cross-Species Comparison in a Tag Cloud Representation

**DOI:** 10.1155/2014/678971

**Published:** 2014-03-31

**Authors:** Jhen-Li Huang, Hao-Teng Chang, Ronshan Cheng, Hui-Huang Hsu, Tun-Wen Pai

**Affiliations:** ^1^Department of Computer Science and Engineering, National Taiwan Ocean University, Keelung 20224, Taiwan; ^2^Graduate Institute of Basic Medical Science, China Medical University, Taichung City 40402, Taiwan; ^3^Department of Computer Science and Information Engineering, Asia University, Taichung City 41354, Taiwan; ^4^Department of Aquaculture, National Taiwan Ocean University, Keelung 20224, Taiwan; ^5^Department of Computer Science and Information Engineering, Tamkang University, New Taipei City 25137, Taiwan

## Abstract

Simple sequence repeats (SSRs) are not only applied as genetic markers in evolutionary studies but they also play an important role in gene regulatory activities. Efficient identification of conserved and exclusive SSRs through cross-species comparison is helpful for understanding the evolutionary mechanisms and associations between specific gene groups and SSR motifs. In this paper, we developed an online cross-species comparative system and integrated it with a tag cloud visualization technique for identifying potential SSR biomarkers within fourteen frequently used model species. Ultraconserved or exclusive SSRs among cross-species orthologous genes could be effectively retrieved and displayed through a friendly interface design. Four different types of testing cases were applied to demonstrate and verify the retrieved SSR biomarker candidates. Through statistical analysis and enhanced tag cloud representation on defined functional related genes and cross-species clusters, the proposed system can correctly represent the patterns, loci, colors, and sizes of identified SSRs in accordance with gene functions, pattern qualities, and conserved characteristics among species.

## 1. Introduction 

Simple sequence repeats (SSRs) are nonrandom distributed nucleotides in genomes of different organisms with repeated basic patterns of lengths from mononucleotide to hexanucleotide [1]. SSRs have been demonstrated as important motifs involved within various biological events including evolutionary processes, gene expression, genetic disease, chromatin organization, and DNA metabolic processes [2–4]. For example, dysplasia disease is a genetic disorder of abnormal cellular development due to imperfect polyalanine expansions (GCC repeats) on* RUNX2 *(*CBFA1*) [5]. Another example of Huntington's disease (HD) was found as an irregular distribution of polyglutamine expansions (CAG repeats) located within the coding regions of Huntingtin (HTT) gene, and the excessive repeat number caused the symptoms of genetic neurological disease which appeared at an earlier stage [6]. In addition to illustrate the effects of mutations and expansions of SSR repeats on diseases, another example to demonstrate the function of SSR motifs is the insulin-like growth factor 1 (*IGF1*) which was confirmed as one of the growth control genes. The* IGF1* gene contains “AC” repeats located within the upstream regions and is a major determinant of small body size in dogs [7–9]. From previous reports, evidences show that SSR regulation relies on pattern of repeat unit, repeat length, and genetic location in the target genes [2]. These features are fundamental parameters for identifying functional SSRs under various biological applications. However, due to abundant amount of SSRs distributed within genome sequences, it is yet challenging to select significant SSR biomarkers or gene regulation related SSRs automatically from limited information. Therefore, identifying highly conserved SSRs through cross-species comparison may provide an alternative approach to recognize significant biomarkers or discover putative gene regulatory SSR motifs from enormous gene candidates under the assumption of natural long-term evolutionary processes. On the other hand, discovering exclusive SSR motifs among different species clusters could be applied as species-specific genetic markers or provide unique genetic functions which were developed after species differentiation events. The comparison of SSR motifs across different species clusters may provide important clues and evidences to further understand evolutionary development.

To efficiently identify SSR biomarkers from large amount of genes in different species, considering a few interested genes at a time provides an intuitive and effective approach. One possible approach of selecting interested gene groups from gene ontology (GO) terms was employed in this study. The GO is a set of structured vocabularies defined by Gene Ontology Consortium [10], which is aimed to provide a universal standard of functional annotation for gene products. All GO terms are connected with each other by directed acyclic graphs with hierarchy relationship. Each term belongs to one of the three independent ontologies: biological process (BP), molecular function (MF), and cellular component (CC) and represent different aspects of gene in temporal, functional, and spatial domains, respectively. In this study, the query biological keywords associated with corresponding GO terms could provide a set of functional-associated gene set for SSR biomarker analysis. Recently, several gene sequence studies associated with GO analysis have been reported, such as the Gene Ontology SSR Hierarchy (GOSH) system which adopts GO terms to reveal prominent orthologous SSR patterns [11], FatiGO which is a web tool for finding significant associations of Gene Ontology terms with groups of genes [2], and Goblet system which performs automatic GO term annotation on anonymous sequences [12].

To enhance the ranking and readability of identified SSR motifs, a tag cloud technique was adopted to display the comparative results of cross-species SSRs. Tag cloud representation is a widespread visualization technology which provides users with an informative image from a designated set of data. Tags of different phases or short sentences represent key information of each entry in the dataset. Multiple tags for various data entries could be displayed in an image simultaneously, and which are manually assigned by users or automatically generated by computer algorithms. Each tag cloud could be shown with different visual attributes such as different sizes or colors. In tradition, different sizes of tags are designed to indicate various levels of representativeness of tags within the dataset [12]. Currently, tag clouds have been widely used in several different types of websites including photo albums, bookmarks, and blogs. It is also used in some tag-based biomedical datasets to help users to rapidly understand the representative information from a complex dataset. For example, the iHOPerator system employed tag cloud technique with related functions for genes analysis [13], INTERFEROME applied tag cloud visualization on gene ontology databases for interferon regulated genes [14], and REVIGO used tag cloud approach to summarize and visualize long lists of gene ontology terms [15]. All these examples have shown that tag cloud visualization techniques could be applied to strengthen key information from complex biological datasets.

In this study, we have collected complete genome sequences of 14 model species as the fundamental dataset. All SSR motifs in each gene were extracted and saved in the designed database in advance. Users can prepare a set of genes or assign keywords to defined query genes and then choose model species of interest for cross-species cluster comparison. Model species of interest could be manually clustered or automatically categorized into two groups of mammal and marine species clusters. SSR retrieval and distribution analysis for single species is also available from the developed system. Once all parameters have been settled, the system will perform online comparison and display all grouped SSR motifs in a tag cloud visualization approach. All significantly conserved or exclusive SSR motifs located within the specified gene sets from two species clusters will be efficiently identified and displayed. In addition, all retrieved SSR biomarker candidates will be shown in a tag cloud representation with occurrence frequency, conserved ratio, gene annotation, sequence contents, and corresponding translated proteins through a fast, responsive, and user-friendly web page design.

## 2. Materials and Methods

### 2.1. System Configuration

In this study, there are fourteen initially selected genomes obtained from Ensembl database [16], and all collected gene sequences with their corresponding gene coordinates and annotations were downloaded for cross-species comparison in next modules. Each gene sequence including upstream and downstream regions was scanned and all perfect/imperfect SSR patterns under different parameter settings were extracted from collected genes and saved in a newly created SSR database. According to gene coordinate information, the developed system determined the corresponding genetic regions for each SSR motif and all related annotations were saved in the same entry. Accordingly, the analytical module utilized cross-species comparison techniques between two assigned species clusters, and all statistically conserved and/or exclusive SSR patterns could be shown under a tag cloud representation technique. All details are introduced in the next two sections.

### 2.2. Genome Sequences

To obtain genome sequences of various organisms, the developed system employed Ensembl release 65 as the major data resource. Ensembl database provides complete genome information on multiple eukaryotic model organisms including whole genome sequence, gene annotations, and molecular functions. To lay emphasis on identification of consensus and unique features of SSRs among different species, two species clusters including fishery and mammal species were initially selected for comparison. Since there were only 6 fishery species that could be collected from Ensembl release 65, we therefore selected another 6 popular mammal species for equivalent status. Besides, two famous research organisms in experimental studies were also included in our database. These intentionally selected model species are zebrafish (*Danio rerio*), stickleback (*Gasterosteus aculeatus*), medaka (*Oryzias latipes*), fugu (*Takifugu rubripes*), tetraodon (*Tetraodon nigroviridis*), and cod (*Gadus morhua*) as fishery species; human (*Homo sapiens*), gorilla (*Gorilla gorilla*), macaque (*Macaca mulatta*), mouse (*Mus musculus*), cow (*Bos taurus*), and dog (*Canis familiaris*) as mammal species; roundworm (*Caenorhabditis elegans*) and fruit fly (*Drosophila melanogaster*) as extra two popular experimental species. These downloaded data include sequence contents, coordinates of exons/introns and UTRs for each gene, and upstream and downstream regions with a length of 2,000 nucleotides.

### 2.3. SSR Motif Database Construction

To accelerate searching speed in identifying all perfect and imperfect orthologous SSRs from a set of specified genes among fourteen species, we performed an autocorrelation based SSR discovery algorithm and constructed the SSR motif database in advance [17]. The autocorrelation algorithm could extract all candidate perfect/imperfect SSR motifs under different threshold parameters through an efficient and effectively approach. In this study, we only considered SSR motifs with nucleotide length longer than 20 nucleotides and the length of fundamental repeat unit ranging from 1 to 6 nucleotides. The SSR motif-searching algorithm also applied a proportional quality factor for defining SSR patterns of different degrees of noise. In this study, three different tolerant settings were tentatively applied for considering noisy patterns within multiscale SSR tag clouds in later presentation. The tolerant parameters were initially set as 0, 0.1, and 0.2 for representing 0, 10, and 20 percent of noisy contents within an SSR motif. The percentage of noise is defined as the ratio of the nonrepeated nucleotides within a total length of an identified SSR, which includes noise types of insertion, deletion, and substitution mutations. In other words, the zero percent noisy rate represents a perfect repeat segment without any tolerance. The formula for the tolerant percentage is shown in the following equation:

(1) $$\begin{array}{c} \text{Tolerant}\;\left(\%\right)=\frac{\text{nonrepeated nucleotides}}{\text{identified SSR length}}\times 100\%. \end{array}$$

An SSR motif could locate in six different genetic regions of a specified gene including coding, intron, 5′ UTR (untranslated region), 3′ UTR, and upstream and downstream regions. In this system, the upstream and downstream regions are defined as an extended range of 2000 nucleotides from the start and end positions of transcription. In addition, according to the shifting mechanism of repeating segments and the complementary based-paired nature in DNA double-stranded helical structures, several possible combinations of SSR patterns could be considered as an identical SSR motif within genetic loci. For example, any rotation of a basic repeat pattern is considered as the same SSR element, such as a “TA” repeat could be also defined as an equivalent repeat motif as an “AT” repeat pattern through one nucleotide shifting. Another situation of an identical SSR motif with different appearance is through complementary based paring and inverse reading from the DNA sequences. For example, the repeat pattern of “AGC” would appear as “GCT” within the other complementary strands of DNA. Therefore, to enumerate all possible SSR patterns in all DNA sequences under these two constraints, there are exactly 501 fundamental basic SSR patterns from 1 to 6 nucleotides in length [18]. However, there is one special condition that should be carefully considered when an SSR motif occurs in coding regions. Since the translation processes convert an mRNA sequence into a string of amino acids through the codon table encoding processes, the equivalent status due to shifting mechanisms and complementary strand should be limited. Here we provide their true translated protein sequences from the locations of identified SSR motifs, and the in-frame information will be clearly annotated when the orthologous repeat motifs are found in coding regions. Finally, to distinguish different SSR patterns from extensive genomic resources, the system defines an identifier for an SSR motif by its basic pattern in accordance with its corresponding genetic location within the specified gene. For example, “AG@Coding” in Ensembl gene id “ENSG00000069329” represents a specific repeated pattern “AG” appearing within the coding region of “ENSG00000069329.” According to prerunning processes under various parameter settings for identifying all possible SSR motifs, in accordance with both detailed coordinates and annotated information from Ensembl database, we constructed a comprehensive SSR motif database for all genes from any specified species. These identified SSR motifs from each gene would be recognized as “tag” items for the following cross-species comparison, and all retrieved SSR tags from the input gene set will be further compared based on occurrence rates and applied to construct a multiscale tag cloud representation.

### 2.4. Grouped Species and Cross-Cluster Comparison

Due to tremendous amount of SSRs nonrandomly distributed in genome sequences, it is not an intuitive task to observe SSR biomarkers or identify gene regulatory related SSR motifs from an individual genome. Hence, we assume the conserved or exclusive SSR motifs providing important clues for identifying functional SSR motifs or representative biomarkers among various species. To emphasize the long-distance relationship from an evolutionary point of view, we have selected two groups of model vertebrate species for orthologous SSR motif comparison. The first group represents the mammalian species including* Bos taurus*,* Canis familiaris*,* Homo sapiens*,* Gorilla gorilla*,* Macaca mulatta*, and* Mus musculus*; the second group represents the fishery species including* Danio rerio*,* Gadus morhua*,* Gasterosteus aculeatus*,* Oryzias latipe*s,* Takifugu rubripes*, and* Tetraodon nigroviridis*. In addition to these twelve clustered species, we also included two widely used model organisms including* Drosophila melanogaster* and* Caenorhabditis elegans*. Nevertheless, in this developed system, users can either apply the previously defined two species groups or manually assign them into two clusters without any limitation. By integrating with cross-species comparison techniques and overrepresentation analysis from assigned gene sets, the SSR patterns with conserved and exclusive characteristics in selected genes between different species clusters can be recognized and treated. An identified conserved SSR motif would be initially defined as an orthologous SSR motif if the conserved ratio meets the minimum threshold in an assigned species cluster. For example, a conserved ratio of 80% denotes the identified conserved SSR pattern that could be found in at least 80% of species in the assigned species cluster, which indicated that there are at least 5 (6∗80% = 4.8) different species possessing the orthologous gene(s) and holding the specific SSR pattern located within the same genetic region among all orthologous gene(s). Regarding the conditions of many-to-many orthologous genes, an SSR motif is defined as holding conserved feature as long as it could be detected in any one of its orthologous genes. The threshold level of conserved ratio can be assigned by users through interactive webpage settings.

Through cross-species comparison between two clustered groups, retrieving conserved or exclusive SSR motifs could help biologists in choosing significant biomarkers from a previously defined gene set before performing biological experiments. On the other hand, exclusive or common SSR motifs between two different species clusters might be regarded as important genetic markers under the evidences of biological evolution and functional conservation.

### 2.5. SSR Tag Cloud Visualization

Tag cloud visualization technique provides keyword representation of text data by showing each tag in various font sizes and colors. To enhance the importance of conserved and exclusive SSR motifs extracted from a set of specified homologous genes between two different species clusters, we adopted the tag cloud representation to display these identified SSR motifs according to their calculated weighting coefficients from query gene sets. In an SSR tag cloud, the tag size of each SSR motif not only indicates the conservation status of the motif among orthologous genes, but also displays the representativeness among different species clusters. A linear accumulation formula and normalization procedures for deciding SSR weighting coefficients were performed for tag size selection. This formula simply counts the number of occurrence times of each SSR motif found from each individual gene in different species clusters. According to the definitions of occurrence rate, if an identified SSR motif is well conserved in two different species clusters or highly represented in the specified gene set, the SSR tag will be assigned with a larger weighting coefficient. Accordingly, the SSR tag will be displayed with a bigger font size in the tag cloud.

**Figure 1:**
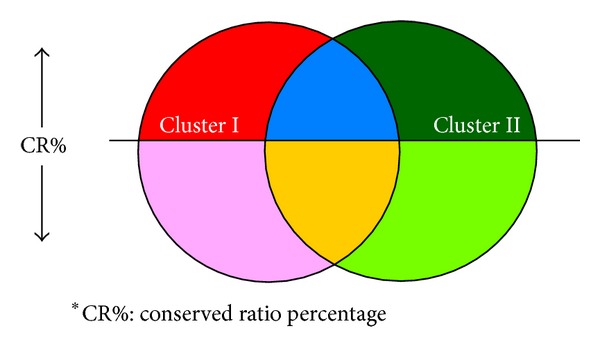
Color coded chart for tag cloud representation of identified SSR motifs between two species clusters and the criterion of conserved ratio.

In order to visually emphasize identified SSR motifs belonging to different species clusters, we applied different colors on SSR tags to distinguish the conserved and/or exclusive features of SSR biomarkers between two species clusters. In this study, red tags represent consensus SSR motifs for the first species cluster only and satisfy the conservation threshold in the first species cluster; pink tags are applied for representing consensus SSR motifs for the first species cluster only, but the conservation threshold is not satisfied; dark green tags represent consensus SSR motifs well conserved within the second species cluster only and these motifs also satisfied the conservation criterion in the second species cluster; light green tags denote consensus SSR motifs in the second species cluster only, but the conservation threshold is not satisfied; blue tags represent the identified SSR patterns well conserved in both species clusters and satisfy the species conservation percentage as well; yellow tags are applied to show identified SSR patterns conserved, but the species conservation criterion is not satisfied for the query gene set from both species clusters. The color-coded information in a resulting tag cloud is shown in Figure 1 and corresponding attributes are described in Table 1. The abbreviated term of CR% represents “conserved ratio” percentage of corresponding species clusters for each simulation.

In the developed system, users can also try to identify imperfect SSR biomarkers by setting different tolerant levels, and the number of retrieved imperfect SSR motifs would be in accordance with the settings proportionally. Higher noisy rates allow more tolerant repeat patterns and reflect larger number of possible SSR motifs. Accordingly, the corresponding tag clouds could be depicted in multiscale representations under various noise threshold settings. In other words, different scales of tag clouds are composed of SSR motifs of different tolerant qualities. For instance, the highest quality of SSR tag cloud represents that all identified conserved SSR motifs are with perfect repeating patterns among different genes and group species. Contrarily, lower quality SSR tag clouds contain more tolerant SSR motifs within the tag image, and which may reflect evolutionary status due to gene specification and/or duplication events from either distant or close species. Multiscale tag clouds provide biologists with an easier way to compare and select suitable SSR candidate motifs as biomarkers through a progressive approach on different tolerance levels, which could be applied in various situations for further design of biological experiments.

## 3. Results

### 3.1. SSR Tag Cloud Web System

**Figure 2:**
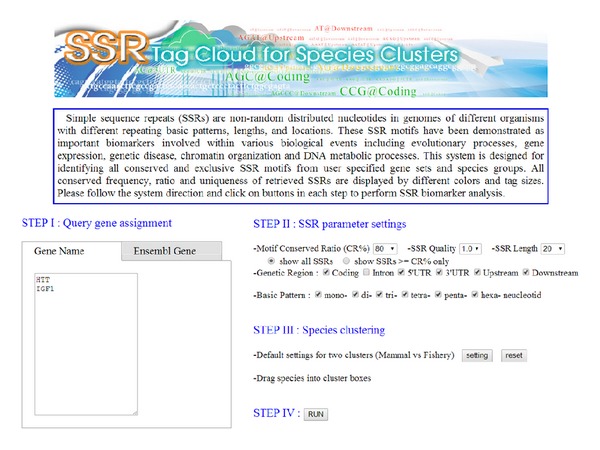
Interface of the SSR tag cloud web system (http://ssrtc.cs.ntou.edu.tw/).

In this study, we have developed an online web system (http://ssrtc.cs.ntou.edu.tw/) for identifying conserved and exclusive SSR biomarkers through cross-species cluster comparison. The main interface of the developed web system is shown in Figure 2. To discover significant SSR biomarker candidates from an automatically generated SSR tag cloud, a user is required to provide gene name(s) or keyword(s) of gene function and simply applies the default parameters for system prediction. In other words, a set of query genes could be defined at the first step by providing relevant EnsemblGene IDs, GO terms, or keywords. Besides, the thresholding settings of SSR feature parameters could also be assigned manually instead of default settings such as genetic region, length of basic pattern, minimum length of SSR motif, SSR quality, species cluster, and SSR motif conserved ratio. The genetic region and length of basic pattern are applied for distinguishing fundamental features of SSR motifs under cross-species cluster comparison. A* minimum SSR length* is applied to define the minimal length for identification of SSR motifs. The* SSR quality factor *represents a tolerance threshold for allowing imperfect SSRs as candidate biomarkers. The developed system initially provides three available settings for efficient identification: 1.0 for perfect SSRs, 0.8 and 0.9 for imperfect SSRs with 20% and 10% tolerant percentages for an identified SSR motif. The function of species cluster assignment is provided for cross-species comparison by classifying species of interest into two clusters. The parameter of* motif conserved ratio* is designed as the percentage of qualified species within a cluster possessing the conserved SSR motif within a target gene. Two different operation modes were designed for the* motif conserved ratio*. If a user chooses the condition of larger than or equal to* motif conserved ratio*, the system will display a resulting SSR tag cloud in 6 colors; otherwise an SSR tag cloud will appear in 3 colors only. Different color modes of an SSR tag cloud are defined in the previous section. Once all parameters and operation modes are defined, the system performs SSR biomarker evaluation automatically and generates a final SSR tag cloud for visualization. The font color of each SSR tag is mainly decided by the* motif conserved ratio* parameter, and the font size depends only on the occurrence frequency of an SSR element. Users can move the mouse device over any SSR item within the resulting tag clouds, and a total appearance number and conserved ratio of the selected SSR motif from the target genes of assigned species cluster will be displayed. The detailed information of each SSR tag is also available in a floating dialog box by clicking on it, which includes Ensembl gene ID, transcript ID of the specified gene possessing the target SSR motif, species name, coordinates in genomes, and DNA sequence contents. Additionally, if an SSR appears within coding regions, then its corresponding protein sequences could be recalled from Ensembl database and shown in an additional window.

### 3.2. SSR Biomarkers for Orthologous Genes

To demonstrate system performance, we have selected all orthologous genes from twelve vertebrate model species (except fruit fly and roundworm). All selected genes possess sequence identities higher than 80% compared to human genome individually. Under this criterion, there are totally 162 orthologous genes selected for the first testing case. If these twelve vertebrate species were classified into two species clusters including mammal and fishery species clusters for comparison, the conserved and exclusive SSR motifs for each gene could be successfully identified and significant SSR biomarker candidates for each individual gene were included in the Supplementary Material available online at http://dx.doi.org/10.1155/2014/678971. Here we only illustrate two genes of ENSG00000069329 and ENSG00000108883 as examples, and all conserved SSR motifs were carefully verified within all orthologous genes from twelve model species.

#### 3.2.1. Case Study of ENSG00000069329 (*VPS35*)

**Figure 3:**
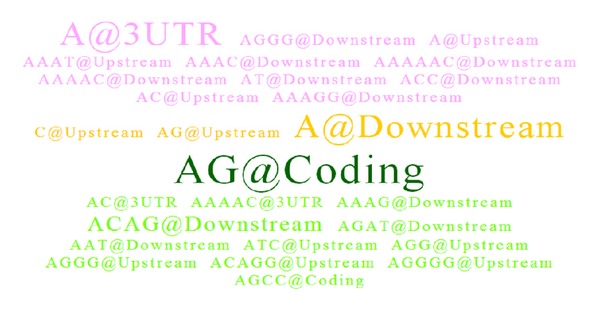
An SSR tag cloud example for ENSG00000069329 (*VPS35*) between two 6-species clusters.

The Ensembl gene ID of ENSG00000069329 is a vacuolar protein sorting gene (*VPS35*) which possesses an average sequence identity of 80% by taking pairwise alignment between human and the other eleven model species. The resulting SSR tag cloud for* VPS35* was shown in Figure 3 by setting* SSR quality* of 80%,* minimum SSR length* of 20 nucleotides, and* motif conserved ratio* of 60% (i.e., required at least 4 species possessing identical SSR motifs in each species cluster). The first* species cluster* was assigned as the mammal group including human, macaque, mouse, cow, dog, and gorilla, and the second* species cluster* was assigned as the fishery group including zebrafish, stickleback, medaka, fugu, tetraodon, and cod. The* genetic region* parameters were set as searching for all regions except introns, and the length of* basic pattern* was selected from 1 to 6 nucleotides for comprehensive representation.

According to Figure 1 for SSR color codes, users can quickly observe that only three coconserved SSR motifs of “C@Upstream,” “AG@Upstream,” and “A@Downstream” in yellow were found between two species clusters. However, in this case, there is not any blue coded SSR tag in this experiment and which implies no coconserved SSR motif existing for at least 4 model species in each species cluster simultaneously. These three yellow color coded SSR tags were found due to their appearance in both species clusters but not well conserved with respect to the assigned conserved ratio. The dark green SSR tag of “AG@Coding” represented the consensus SSR motif could be found only in the second cluster of fishery species with more than 4 fishery species containing the SSR motif at coding region, but this motif pattern at coding region was not found in any mammal species from the first cluster. The light green SSR tags represented consensus SSR motifs which were found only in the fishery group but do not satisfied the* motif conserved ratio* requirement of 80%; that is, these light green coded SSR patterns were only found with less than 4 fishery species. On the other hand, the pink coded SSR tags represented consensus SSR motifs found only in the mammal species cluster exclusively with less than 4 mammal species. In addition, the dark green SSR tag of “AG@Coding” with the biggest font size implied this SSR holding as the most representative and exclusive feature for fishery species compared to mammal species.

#### 3.2.2. Case Study of ENSG00000108883 (*EFTUD2*)

**Figure 4:**
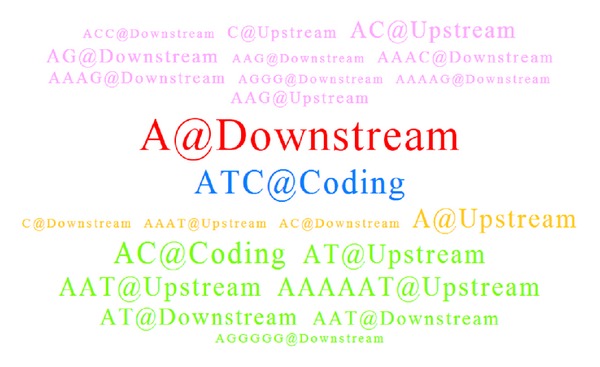
An SSR tag cloud example for ENSG000000108883 (*EFTUD2*) between two 6-species clusters.

The Ensemble gene ID of ENSG00000108883 is an elongation factor Tu GTP binding domain (*EFTUD2*) which possesses an average sequence identity of 80% by taking pairwise alignment between human species and other 11 model species individually. The resulting SSR tag cloud for* EFTUD2* was shown in Figure 4 by setting exactly the same parameters as the previous example. According to the resulting tag cloud, users can immediately identified that only one coconserved SSR tag of “ATC@Coding” could be found as a notable biomarker between two species clusters and it was well conserved across at least 4 species in each species cluster. Hence, the SSR tag was indicated by blue. Furthermore, one red coded SSR tag of “A@Downstream” represented the consensus SSR motifs found only in the first mammal species cluster and more than 4 species containing the SSR motif at coding region. However, this motif could not be found in any fishery species. The pink SSR tags represented all conserved SSR motifs found only in the mammal group but not satisfied the requirement of* Motif Conserved Ratio*. Similarly, the light green coded SSR tags represented consensus SSR motifs only found in the fishery species cluster exclusively with less than 4 fishery species. In addition, the red SSR tag of “A@Downstream” was shown with the biggest font size, which implied the SSR holding as the most representative and exclusive for mammal species compared to all other SSR candidates.

Interestingly, the first gene,* VPS35* (ENSG00000069329), is associated with “Parkinson's disease (PD)” [19], and the second gene,* EFTUD2* (ENSG00000108883), causes “mandibulofacial dysostosis with microcephaly” [20]. In both cases, so far, scientists have only demonstrated that both diseases were caused by some gene mutations. Through* in silico* SSR biomarker detection by our proposed system, we could efficiently identify many important conserved and exclusive SSRs between two grouped species as biomarkers. However, without experimental verification, we could not make sure whether both diseases possess a true correlation with identified SSR motifs. To gain more confidence on the proposed system, we verified on some disease genes which were known to be associated with some specific SSR biomarkers. If a genetic disease is indeed caused by abnormal distributions of SSR motifs, we expect that our proposed SSR tag cloud representation system could identify those significant SSR biomarkers in an efficient and effective way.

### 3.3. Case Study of a Set of Skeletal Development Genes

To demonstrate functionally related SSR motifs, we have selected a gene set containing specific function of skeletal development. A total of 17 genes associated with such function are selected and these genes are* HOXA11*,* ZIC2*,* ALX4*,* HOXA2*,* DLX2*,* HOXA7*,* TWIST1*,* HOXC13*,* RUNX2*,* SOX9*,* HOXD11*,* HOXD13*,* GDF11*,* HLX*,* SIX3*,* HOXD8*, and* HOXA10* [21]. In this example, we have shown that the detailed information of each SSR tag is available in a floating dialog by clicking on it, and the appearance number and conserved ratio of a selected SSR motif from the target genes can be viewed by moving mouse cursor over the SSR tag.

**Figure 5:**
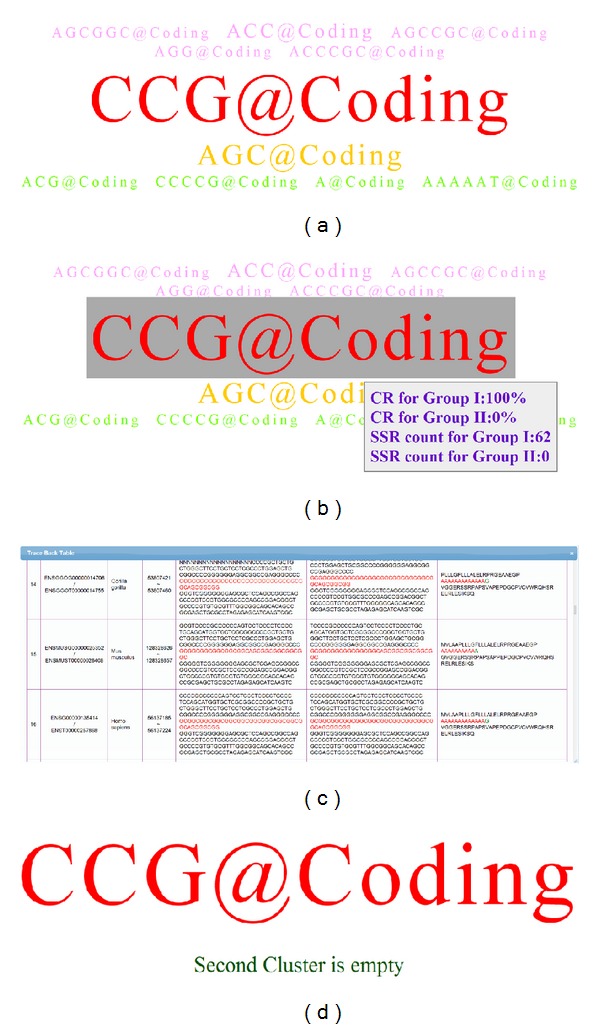
(a) SSR tag cloud for 17 skeletal development related genes constrained to coding regions; (b) results of moving a mouse device over the SSR tag of “CCG@Coding”; (c) detailed information of the SSR tag “CCG@Coding” in a floating dialog; (d) an SSR tag cloud for 17 skeletal development related genes by showing SSRs possessing high conserved ratios only.

The resulting SSR tag clouds from different combinatorial settings for 17 skeletal development related genes were shown in Figure 5. In Figure 5(a), the parameter settings were defined as follows:* SSR quality* of 90% for perfect SSR patterns, minimum* SSR length* of 20 nucleotides,* motif conserved ratio* of 80% (i.e., at least 5 species possessing identical SSR motifs in each species cluster), and all possible SSR candidates were shown. The first* species cluster *was assigned as the mammal group including human, macaque, mouse, cow, dog, and gorilla; the second* species cluster *was assigned as the fishery group including zebrafish, stickleback, medaka, fugu, tetraodon, and cod. The filter of* genetic region* was selected for coding region only, and the length of* basic pattern* was selected from 1 to 6 nucleotides for comprehensive representation. According to these settings, the simulated results were shown in Figure 5: the red coded SSR tag of “CCG@Coding” represented the only exclusive SSR motifs well conserved in mammal species. This tag could be found from at least 5 species within the mammal group, and it is highly correlated to the skeletal development related genes. Users can move a mouse device over the tag of “CCG@Coding,” and the appearance number and conserved ratio of the selected SSR motif would be shown with a pop-up icon. In Figure 5(b), the CCG@Coding motifs appear in the mammal species cluster with a total of 62 times and a conserved ratio of 100%, while no such an SSR motif could be discovered from the skeletal development gene set within the fishery species cluster. If a user clicked on the tag of “CCG@Coding,” detailed information of the SSR tag will be shown by a floating dialog with Ensembl gene ID, transcript ID, species name, coordinates in genomes, and DNA sequence contents. Particularly, if the SSRs appeared within coding regions, the table also provided the detailed information of cDNA sequence and its corresponding translated protein sequences. In Figure 5(c), the CCG repeated pattern in the last row of human's ENSG00000135414 (*GDF11*) gene is located at chromosome 12 and its coordinates are from 56137185 to 56137224. Since the CCG repeated pattern was found in coding regions, the table also provided the detailed information of DNA, cDNA, and corresponding protein sequence contents. Actually, this repeated pattern in* RUNX2* gene at coding region is a polyalanine peptide (GCC repeat in coding region), and it indeed plays a crucial role in cellular development function. Abnormal distribution of this polyalanine repeat biomarker might cause dysplasia disease, a genetic disorder of abnormal cellular development.

In Figure 5(d), most of parameters were set identically as Figure 5(a), except the display parameter was modified for showing highly conserved SSRs instead of showing all of identified SSRs. In the other words, tags with pink, light green, and yellow color codes would be hidden. The corresponding tag showed only one red coded tag of “CCG@Coding” existed under such high conservation requirements. Again, the SSR motif of “CCG@Coding” represented as a significant biomarker in mammal species highly correlated to the skeletal development related genes.

### 3.4. Case Study of Gene Ontology Term of “Embryonic Cranial Skeleton Morphogenesis”

**Figure 6:**
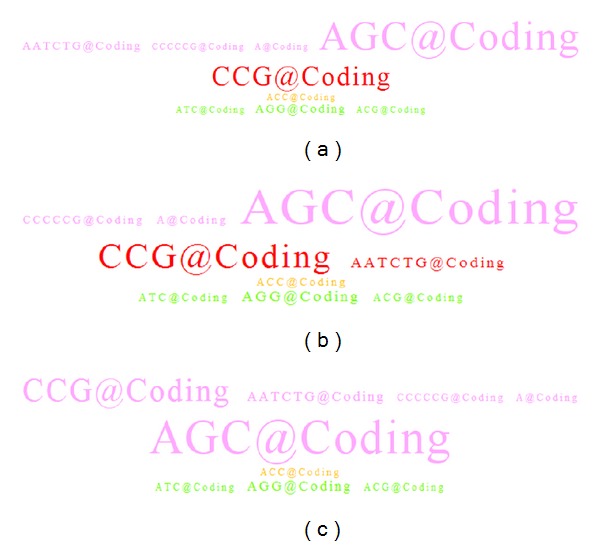
(a) SSR tag cloud for GO keyword “embryonic cranial skeleton morphogenesis” with motif conserved ratio of 80%; (b) motif conserved ratio of 60%; (c) motif conserved ratio of 100%.

To demonstrate functionally related SSR motifs through GO term assignment, we selected a GO term of “embryonic cranial skeleton morphogenesis.” The related genes annotated by this GO term include* TBX15, SIX4, DLX2, PRRX1, TWIST1, BMP4, SIX1, SMAD2, NIPBL, NODAL, WNT9B, TGFBR2, GAS1, SIX2, FOXC2, SMAD3, TBX1, TGFBR2, TBX15, GNAS, PRRX2, TGFBR1, TFAP2A, SMAD2, SETD2, BMP4, SMAD3, TWIST2, TFAP2A, SMAD3,* TGFBR1, and* BMP4*. To compare and show different results by various settings, we have tried several combinations of input parameters which were different from system default settings. In this case study, the parameter settings were defined as follows:* SSR quality* of 90% for perfect SSR patterns,* minimum SSR length* of 20 nucleotides,* motif conserved ratio* of 80% (i.e., at least 5 species possessing identical SSR motifs in each species cluster) and showed all possible SSR candidates. The first* species cluster *was assigned as mammal group and the second* species cluster *for fishery group as default settings. The filter of* genetic region* was selected for analyzing on coding regions only, and the length of* basic pattern* was selected from 1 to 6 nucleotides. According to these settings, the simulated results were shown in Figure 6(a). We could observe that there was only one red color coded SSR tag of “CCG@Coding” and which is the unique biomarker conserved in mammal species with respect to the embryonic cranial skeleton morphogenesis related genes.

Then, we lowered down the* motif conserved ratio* to 60% and the resulting SSR tag cloud was shown in Figure 6(b). We could observe that several tags were changed by their coded colors. Taking red color coded tags as an example, there was only one red tag “CCG@Coding” in previous Figure 6(a), but in Figure 6(b), we noticed that the red color coded SSR tags increased another tag of “AATCTG@Coding” which was displayed in originally denoted as pink in Figure 6(a). Inversely, if we increased the* motif conserved ratio* to 100%, the result was shown in Figure 6(c) with no red color coded SSR tag in this cloud. Compared to Figure 6(a), the original red tag of “CCG@Coding” was changed into pink due to only 5 out of 6 species in the mammal group holding the tag of “CCG@Coding.” In both Figures 6(b) and 6(c), we simply observed that color coded tags may switch their colors through different* motif conserved ratio *adjustments. The higher setting of* motif conserved ratio* reduces the amount of red, green, and blue color coded tags.

### 3.5. An Example of Genetic Disease of “Huntington's Disease (HD)”

To demonstrate genetic diseases caused by abnormal distribution of SSR motifs, we have selected a well-known neurodegenerative genetic disease “Huntington's disease (HD)” as an example. HD was found as an irregular distribution of polyglutamine expansions (CAG repeats) located within the coding regions of ENSG00000197386 (*HTT*) gene at chromosome 4 [22]. It appears with involuntary movements caused by losing muscle coordination and leads to psychiatric problems. The nucleotide repeat length and the average age of symptom occurrence of Huntington's disease were in inverse relationship [23].

**Figure 7:**
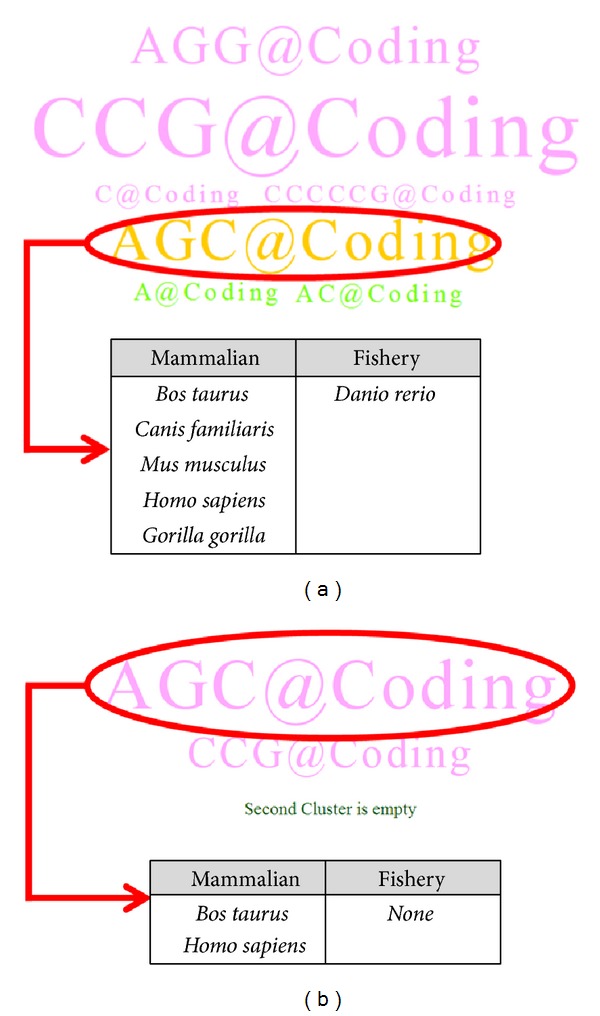
(a) SSR tag cloud for HTT gene with SSR quality of 80%, Motif Conserved Ratio of 80%, and 5 organisms holding the conserved SSR tag of “AGC@Coding”; (b) SSR quality of 100%, Motif Conserved Ratio of 80%, and only two species of human and cattle species holding the perfect SSR tag of “AGC@Coding”.

The verification results of SSR tag cloud were shown in Figure 7, and the parameter settings were defined as follows:* SSR quality* of 100% and 80%,* minimum SSR length* of 20 nucleotides,* motif conserved ratio* of 80% (i.e., at least 5 species possessing identical SSR motifs in each species cluster), and with a selection of “show all SSRs”. The first* species cluster* was assigned as mammal group while the second* species cluster* as fishery group. In Figure 7, we could observe the “AGC@Coding” in both two-tag clouds as an important biomarker. In fact, according to shifting transformation of SSR repeat pattern, the “AGC” repeat unit could be theoretically considered as the same pattern of “CAG” for efficient identification. However, SSRs located within coding regions would be further translated into their corresponding amino acid sequences according to precise loci verification on exon regions. Frame shifted SSRs in coding regions might result in different coded amino acids. For example, the coded amino acid of the trinucleotide pattern of “AGC” is serine(S) and “CAG” for glutamine (Q). Therefore, identified SSRs in coding regions should be carefully treated and translated into an appropriated protein sequence based on annotated genome database. In this example, we noticed that a significant SSR motif of “AGC@Coding” in HTT genes could be identified with different sizes (occurrence rates) according to various* SSR quality* settings. This repeat motif in coding regions appears in most mammal species except macaque with a minimum length requirement of 20 nucleotides. Besides, only zebrafish possesses a similar repeat motif in coding region among all fishery species. When the parameter of* SSR quality* was increased to 100% (without any tolerance), the pattern of “AGC@Coding” (or equivalently to “CAG@Coding” in DNA sense strand) could be retrieved from both cattle and human in mammal species only. We could observe that the font size and color of each SSR tag were gradually changed according to different settings of tolerance rate. Accordingly, the tag of “AGC@Coding” appeared with the biggest icon in pink when compared to all other SSRs in coding regions, and it reflected the significance of exclusive features for mammal species compared to fishery species. These observations might also provide important information for biologists for animal species selection in future experimental studies regarding specific diseases.

## 4. Discussion

**Figure 8:**
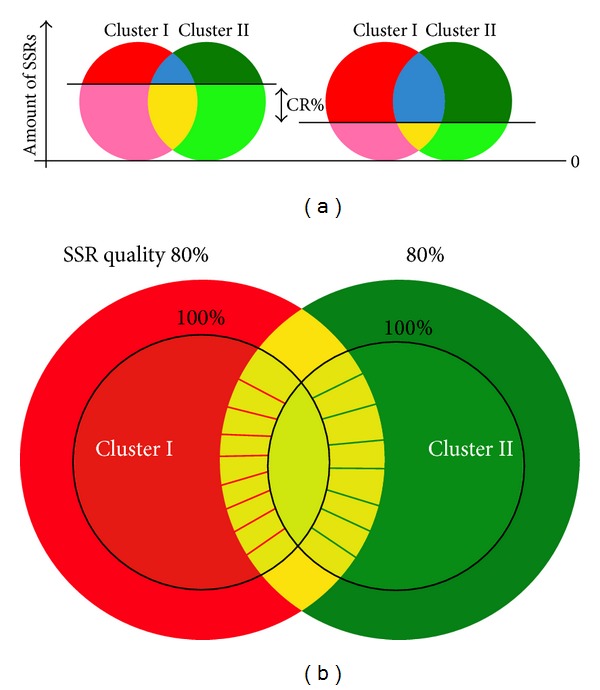
(a) Relationship between the parameter of* motif conserved ratio* and the amount of SSR tags in different colors; (b) relationship between the parameter of* SSR quality* and SSR tag colors.

Two key parameters affect the color and size distribution within an SSR tag cloud. The first one is the* motif conserved ratio*. Different conserved ratio values change colors of SSR tags. When the* motif conserved ratio* increased, the amount of red, green, and blue tags might decrease. In Figure 8(a), Cluster I represents the first* species cluster *and Cluster II represents the second* species cluster*. The horizontal straight line in the figure represents a* motif conserved ratio* value. When the CR% threshold value is increased, the areas of red, blue, and dark green decreased. In contrast, when the CR% threshold value is decreased, the areas of red, blue, and dark green increased. The area is proportional to the amount of SSR tags.

The second important parameter for a tag cloud is the* SSR quality* threshold. As shown in Figure 8(b), different* SSR quality* values were not only changing the number of SSR tags but also transforming the colors. Increment of* SSR quality* value may reduce the amount of SSR tags, since the SSRs with higher qualities are always a subset of SSRs with lower qualities. When a quality threshold decreases to gain more SSR candidates, part of red and green tags might change their colors into yellow or blue tags, respectively. This is mainly caused by newly intersecting region after expanding SSR candidates.

Besides, a few common SSR tags originally coded in yellow might be transformed into either red or green through increasing the quality factors, which is mainly because the total number of species possessing certain SSR tag is decreased, and therefore the conserved SSR motifs between two species clusters might become representative SSR tags for one species cluster exclusively. In Table 2, a list of total amount of SSR motifs for each species is presented by setting a minimum* SSR length* of 20 nucleotides. The SSR quantities for mammal species are usually more than fishery species, and the increment of* SSR quality* value reduces the amount of SSR motifs in each species generally.

## 5. Conclusion

SSRs are nonrandomly distributed nucleotides in the genomes with repeating basic patterns of lengths from 1 to 6 nucleotides, and a large number of functional SSR motifs have been demonstrated as important biomarkers involved within various biological processes and gene regulations. Due to abundant number of SSRs in each species genomes, it is difficult to recognize significant SSR biomarkers or gene regulation related SSRs mainly based on repeat sequence length, genetic locations, and fundamental repeat pattern of an SSR motif. In this paper, we proposed the concept of identifying SSR biomarker candidate through cross-species cluster comparison on a specified set of target genes. The developed system provides an online tool with multiparameter selection functions, and the identified SSR motifs are displayed by a tag cloud visualization method. The exclusive and consensus SSR motifs between two species clusters are shown in different font colors and sizes in an efficient approach. The* in silico *comparison of SSR motifs across different species clusters may provide the clues and evidences for further understanding of evolutionary development and functional associations.

## Supplementary Material

The Supplementary Material provides an orthologous gene list which contains 162 genes from 12 selected model species. Each gene possesses a sequence identity higher than 80% compared to its corresponding orthologous gene in human genome respectively.

## Figures and Tables

**Table 1:** Relationship between colors, species clusters, and conserved ratios of detected SSR motifs.

Color	Species cluster	Conserved motif ratio
Red	I	≥CR%
Pink	I	<CR%
Blue	I and II	≥CR%
Yellow	I and II	<CR%
Dark green	II	≥CR%
Light green	II	<CR%

**Table 2:** The number of SSR motifs of each species for various *SSR  quality* settings.

Scientific name	Species name	*SSR quality* 80%	*SSR quality* 90%	*SSR quality* 100%
*Danio rerio *	Zebrafish	1,175,832	594,741	401,503
*Gasterosteus aculeatus *	Stickleback	160,413	87,343	51,779
*Oryzias latipes *	Medaka	122,505	37,730	15,460
*Takifugu rubripes *	Fugu	261,612	148,043	90,753
*Tetraodon nigroviridis *	Tetraodon	119,557	69,473	43,584
*Gadus morhua *	Cod	359,592	209,540	123,880

*Homo sapiens *	Human	3,023,284	1,406,186	644,338
*Gorilla gorilla *	Gorilla	757,571	344,973	152,403
*Macaca mulatta *	Macaque	1,075,737	526,515	225,403
*Mus musculus *	Mouse	2,463,222	1,301,019	812,873
*Bos taurus *	Cow	323,386	132,923	44,906
*Canis familiaris *	Dog	715,776	340,433	152,502
*Caenorhabditis elegans *	Roundworm	59,273	13,637	4,225
*Drosophila melanogaster *	Fruit fly	199,458	79,952	21,223
