# Supplementary material for: Identification of Simple Sequence Repeat Biomarkers through Cross-Species Comparison in a Tag Cloud Representation
Source: Biomed Res Int. 2014 Mar 31;2014:678971. doi: 10.1155/2014/678971 (PMC3988901; doi:10.1155/2014/678971)
Supplement: Supplementary file 1 — The Supplementary Material provides an orthologous gene list which contains 162 genes from 12 selected model species. Each gene possesses a sequence identity higher than 80% compared to its corresponding orthologous gene in human genome respectively. [file 678971.f1.pdf]

Supplementary Document

Table S1: An orthologous gene list containing 162 genes from 12 selected model species. Each gene possesses a sequence identity higher than 80% compared to human genome mutually.

Ensembl Gene ID	Gene Name	Description
ENSG00000006042	*TMEM98*	transmembrane protein 98
ENSG00000013016	*EHD3*	EH-domain containing 3
ENSG00000025800	*KPNA6*	karyopherin alpha 6 (importin alpha 7)
ENSG00000044574	*HSPA5*	heat shock 70kDa protein 5 (glucose-regulated protein, 78kDa)
ENSG00000047249	*ATP6V1H*	ATPase, H+ transporting, lysosomal 50/57kDa, V1 subunit H
ENSG00000048540	*LMO3*	LIM domain only 3 (rhombotin-like 2)
ENSG00000054803	*CBLN4*	cerebellin 4 precursor
ENSG00000055163	*CYFIP2*	cytoplasmic FMR1 interacting protein 2
ENSG00000061676	*NCKAP1*	NCK-associated protein 1
ENSG00000061918	*GUCY1B3*	guanylate cyclase 1, soluble, beta 3
ENSG00000062725	*APPBP2*	amyloid beta precursor protein (cytoplasmic tail) binding protein 2
ENSG00000066117	*SMARCD1*	SWI/SNF related, matrix associated, actin dependent regulator of chromatin, subfamily d, member 1
ENSG00000066777	*ARFGEF1*	ADP-ribosylation factor guanine nucleotide-exchange factor 1 (brefeldin A-inhibited)
ENSG00000068793	*CYFIP1*	cytoplasmic FMR1 interacting protein 1
ENSG00000069329	*VPS35*	vacuolar protein sorting 35 homolog (S. cerevisiae)
ENSG00000070718	*AP3M2*	adaptor-related protein complex 3, mu 2 subunit

ENSG00000073712	*FERMT2*	fermitin family member 2
ENSG00000075290	*WNT8B*	wingless-type MMTV integration site family, member 8B
ENSG00000077147	*TM9SF3*	transmembrane 9 superfamily member 3
ENSG00000077458	*FAM76B*	family with sequence similarity 76, member B
ENSG00000077549	*CAPZB*	capping protein (actin filament) muscle Z-line, beta
ENSG00000078142	*PIK3C3*	phosphoinositide-3-kinase, class 3
ENSG00000079785	*DDX1*	DEAD (Asp-Glu-Ala-Asp) box polypeptide 1
ENSG00000084623	*EIF3I*	eukaryotic translation initiation factor 3, subunit I
ENSG00000086475	*SEPHS1*	selenophosphate synthetase 1
ENSG00000087191	*PSMC5*	proteasome (prosome, macropain) 26S subunit, ATPase, 5
ENSG00000100206	*DMC1*	DMC1 dosage suppressor of mck1 homolog, meiosis-specific homologous recombination (yeast)
ENSG00000100220	*C22orf28*	chromosome 22 open reading frame 28
ENSG00000100280	*AP1B1*	adaptor-related protein complex 1, beta 1 subunit
ENSG00000100353	*EIF3D*	eukaryotic translation initiation factor 3, subunit D
ENSG00000100410	*PHF5A*	PHD finger protein 5A
ENSG00000100528	*CNIH*	cornichon homolog (Drosophila)
ENSG00000100796	*SMEK1*	SMEK homolog 1, suppressor of mek1 (Dictyostelium)
ENSG00000100934	*SEC23A*	Sec23 homolog A (S. cerevisiae)
ENSG00000101146	*RAE1*	RAE1 RNA export 1 homolog (S. pombe)
ENSG00000101310	*SEC23B*	Sec23 homolog B (S. cerevisiae)
ENSG00000101460	*MAP1LC3A*	microtubule-associated protein 1 light chain 3 alpha

ENSG00000102978	*POLR2C*	polymerase (RNA) II (DNA directed) polypeptide C, 33kDa
ENSG00000103043	*VAC14*	Vac14 homolog (S. cerevisiae)
ENSG00000105258	*POLR2I*	polymerase (RNA) II (DNA directed) polypeptide I, 14.5kDa
ENSG00000105443	*CYTH2*	cytohesin 2
ENSG00000105618	*PRPF31*	PRP31 pre-mRNA processing factor 31 homolog (S. cerevisiae)
ENSG00000107105	*ELAVL2*	ELAV (embryonic lethal, abnormal vision, Drosophila)-like 2 (Hu antigen B)
ENSG00000108055	*SMC3*	structural maintenance of chromosomes 3
ENSG00000108061	*SHOC2*	soc-2 suppressor of clear homolog (C. elegans)
ENSG00000108306	*FBXL20*	F-box and leucine-rich repeat protein 20
ENSG00000108379	*WNT3*	wingless-type MMTV integration site family, member 3
ENSG00000108591	*DRG2*	developmentally regulated GTP binding protein 2
ENSG00000108883	*EFTUD2*	elongation factor Tu GTP binding domain containing 2
ENSG00000109956	*B3GAT1*	beta-1,3-glucuronyltransferase 1 (glucuronosyltransferase P)
ENSG00000110367	*DDX6*	DEAD (Asp-Glu-Ala-Asp) box polypeptide 6
ENSG00000110906	*KCTD10*	potassium channel tetramerisation domain containing 10
ENSG00000111530	*CAND1*	cullin-associated and neddylation-dissociated 1
ENSG00000111707	*SUDS3*	suppressor of defective silencing 3 homolog (S. cerevisiae)
ENSG00000111783	*RFX4*	regulatory factor X, 4 (influences HLA class II expression)
ENSG00000112237	*CCNC*	cyclin C
ENSG00000112282	*MED23*	mediator complex subunit 23
ENSG00000112308	*C6orf62*	chromosome 6 open reading frame 62

ENSG00000112333	*NR2E1*	nuclear receptor subfamily 2, group E, member 1
ENSG00000112335	*SNX3*	sorting nexin 3
ENSG00000113369	*ARRDC3*	arrestin domain containing 3
ENSG00000113595	*TRIM23*	tripartite motif containing 23
ENSG00000113719	*ERGIC1*	endoplasmic reticulum-golgi intermediate compartment (ERGIC) 1
ENSG00000114030	*KPNA1*	karyopherin alpha 1 (importin alpha 5)
ENSG00000114388	*NPRL2*	nitrogen permease regulator-like 2 (S. cerevisiae)
ENSG00000115866	*DARS*	aspartyl-tRNA synthetase
ENSG00000116586	*LAMTOR2*	late endosomal/lysosomal adaptor, MAPK and MTOR activator 2
ENSG00000117153	*KLHL12*	kelch-like 12 (Drosophila)
ENSG00000117505	*DR1*	down-regulator of transcription 1, TBP-binding (negative cofactor 2)
ENSG00000119487	*MAPKAP1*	mitogen-activated protein kinase associated protein 1
ENSG00000119820	*YIPF4*	Yip1 domain family, member 4
ENSG00000120509	*PDZD11*	PDZ domain containing 11
ENSG00000121022	*COPS5*	COP9 constitutive photomorphogenic homolog subunit 5 (Arabidopsis)
ENSG00000123159	*GIPC1*	GIPC PDZ domain containing family, member 1
ENSG00000123353	*ORMDL2*	ORM1-like 2 (S. cerevisiae)
ENSG00000123374	*CDK2*	cyclin-dependent kinase 2
ENSG00000123395	*C12orf44*	chromosome 12 open reading frame 44
ENSG00000123908	*EIF2C2*	eukaryotic translation initiation factor 2C, 2
ENSG00000124207	*CSE1L*	CSE1 chromosome segregation 1-like (yeast)

ENSG00000124209	*RAB22A*	RAB22A, member RAS oncogene family
ENSG00000125037	*TMEM111*	transmembrane protein 111
ENSG00000125354	*SEPT6*	septin 6
ENSG00000125820	*NKX2-2*	NK2 homeobox 2
ENSG00000125851	*PCSK2*	proprotein convertase subtilisin/kexin type 2
ENSG00000126970	*ZC4H2*	zinc finger, C4H2 domain containing
ENSG00000127054	*CPSF3L*	cleavage and polyadenylation specific factor 3-like
ENSG00000127774	*TMEM93*	transmembrane protein 93
ENSG00000127922	*SHFM1*	split hand/foot malformation (ectrodactyly) type 1
ENSG00000128699	*ORMDL1*	ORM1-like 1 (S. cerevisiae)
ENSG00000129083	*COPB1*	coatomer protein complex, subunit beta 1
ENSG00000129562	*DAD1*	defender against cell death 1
ENSG00000130520	*LSM4*	LSM4 homolog, U6 small nuclear RNA associated (S. cerevisiae)
ENSG00000132142	*ACACA*	acetyl-CoA carboxylase alpha
ENSG00000132356	*PRKAA1*	protein kinase, AMP-activated, alpha 1 catalytic subunit
ENSG00000132640	*BTBD3*	BTB (POZ) domain containing 3
ENSG00000132646	*PCNA*	proliferating cell nuclear antigen
ENSG00000132912	*DCTN4*	dynactin 4 (p62)
ENSG00000133119	*RFC3*	replication factor C (activator 1) 3, 38kDa
ENSG00000134014	*ELP3*	elongation protein 3 homolog (S. cerevisiae)
ENSG00000134058	*CDK7*	cyclin-dependent kinase 7
ENSG00000135932	*CAB39*	calcium binding protein 39

ENSG00000136518	*ACTL6A*	actin-like 6A
ENSG00000137522	*RNF121*	ring finger protein 121
ENSG00000137822	*TUBGCP4*	tubulin, gamma complex associated protein 4
ENSG00000137947	*GTF2B*	general transcription factor IIB
ENSG00000140612	*SEC11A*	SEC11 homolog A (S. cerevisiae)
ENSG00000140829	*DHX38*	DEAH (Asp-Glu-Ala-His) box polypeptide 38
ENSG00000141429	*GALNT1*	UDP-N-acetyl-alpha-D-galactosamine:polypeptide N-acetylgalactosaminyltransferase 1 (GalNAc-T1)
ENSG00000144043	*TEX261*	testis expressed 261
ENSG00000144231	*POLR2D*	polymerase (RNA) II (DNA directed) polypeptide D
ENSG00000144580	*RQCD1*	RCD1 required for cell differentiation1 homolog (S. pombe)
ENSG00000144935	*TRPC1*	transient receptor potential cation channel, subfamily C, member 1
ENSG00000145817	*YIPF5*	Yip1 domain family, member 5
ENSG00000145833	*DDX46*	DEAD (Asp-Glu-Ala-Asp) box polypeptide 46
ENSG00000147164	*SNX12*	sorting nexin 12
ENSG00000148606	*POLR3A*	polymerase (RNA) III (DNA directed) polypeptide A, 155kDa
ENSG00000148943	*LIN7C*	lin-7 homolog C (C. elegans)
ENSG00000149100	*EIF3M*	eukaryotic translation initiation factor 3, subunit M
ENSG00000151348	*EXT2*	exostosin 2
ENSG00000151729	*SLC25A4*	solute carrier family 25 (mitochondrial carrier; adenine nucleotide translocator), member 4
ENSG00000154473	*BUB3*	budding uninhibited by benzimidazoles 3 homolog (yeast)
ENSG00000157837	*SPPL3*	Signal peptide peptidase-like 3

ENSG00000158864	*NDUFS2*	NADH dehydrogenase (ubiquinone) Fe-S protein 2, 49kDa (NADH-coenzyme Q reductase)
ENSG00000159210	*SNF8*	SNF8, ESCRT-II complex subunit, homolog (S. cerevisiae)
ENSG00000160563	*MED27*	mediator complex subunit 27
ENSG00000162374	*ELAVL4*	ELAV (embryonic lethal, abnormal vision, Drosophila)-like 4 (Hu antigen D)
ENSG00000162409	*PRKAA2*	protein kinase, AMP-activated, alpha 2 catalytic subunit
ENSG00000163479	*SSR2*	signal sequence receptor, beta (translocon-associated protein beta)
ENSG00000163625	*WDFY3*	WD repeat and FYVE domain containing 3
ENSG00000163636	*PSMD6*	proteasome (prosome, macropain) 26S subunit, non-ATPase, 6
ENSG00000164091	*WDR82*	WD repeat domain 82
ENSG00000164167	*LSM6*	LSM6 homolog, U6 small nuclear RNA associated (S. cerevisiae)
ENSG00000164332	*UBLCP1*	ubiquitin-like domain containing CTD phosphatase 1 [
ENSG00000164576	*SAP30L*	SAP30-like
ENSG00000165630	*PRPF18*	PRP18 pre-mRNA processing factor 18 homolog (S. cerevisiae)
ENSG00000166407	*LMO1*	LIM domain only 1 (rhombotin 1)
ENSG00000166747	*AP1G1*	adaptor-related protein complex 1, gamma 1 subunit
ENSG00000166887	*VPS39*	vacuolar protein sorting 39 homolog (S. cerevisiae)
ENSG00000167965	*MLST8*	MTOR associated protein, LST8 homolog (S. cerevisiae)
ENSG00000168291	*PDHB*	pyruvate dehydrogenase (lipoamide) beta
ENSG00000168301	*KCTD6*	potassium channel tetramerisation domain containing 6
ENSG00000168385	*SEPT2*	septin 2
ENSG00000169504	*CLIC4*	chloride intracellular channel 4

ENSG00000170312	*CDK1*	cyclin-dependent kinase 1
ENSG00000172318	*B3GALT1*	UDP-Gal:betaGlcNAc beta 1,3-galactosyltransferase, polypeptide 1
ENSG00000173418	*NAA20*	N(alpha)-acetyltransferase 20, NatB catalytic subunit
ENSG00000174231	*PRPF8*	PRP8 pre-mRNA processing factor 8 homolog (S. cerevisiae)
ENSG00000175087	*PDIK1L*	PDLIM1 interacting kinase 1 like
ENSG00000178307	*TMEM11*	transmembrane protein 11
ENSG00000181852	*RNF41*	ring finger protein 41
ENSG00000183207	*RUVBL2*	RuvB-like 2 (E. coli)
ENSG00000183475	*ASB7*	ankyrin repeat and SOCS box containing 7
ENSG00000183726	*TMEM50A*	transmembrane protein 50A
ENSG00000185009	*AP3M1*	adaptor-related protein complex 3, mu 1 subunit
ENSG00000185527	*PDE6G*	phosphodiesterase 6G, cGMP-specific, rod, gamma
ENSG00000185722	*ANKFY1*	ankyrin repeat and FYVE domain containing 1
ENSG00000187555	*USP7*	ubiquitin specific peptidase 7 (herpes virus-associated)
ENSG00000196655	*TRAPPC4*	trafficking protein particle complex 4
ENSG00000198700	*IPO9*	importin 9
ENSG00000203879	*GDI1*	GDP dissociation inhibitor 1
ENSG00000204628	*GNB2L1*	guanine nucleotide binding protein (G protein), beta polypeptide 2-like 1
ENSG00000213246	*SUPT4H1*	suppressor of Ty 4 homolog 1 (S. cerevisiae)
